# From occasional records to regular presence: Increasing occurrence of the bastard grunt *Pomadasys incisus* in the French Ligurian Sea (northwestern Mediterranean)

**DOI:** 10.1111/jfb.70488

**Published:** 2026-05-04

**Authors:** Astruch Patrick, Héritier David, Serre Christophe, Strebler Yann, Schohn Thomas, Belloni Bruno, Le Diréach Laurence

**Affiliations:** ^1^ GIS Posidonie, Aix‐Marseille University, OSU Pytheas Marseille Cedex 9 France; ^2^ Esterel Côte d'Azur Agglomération Saint Raphaël France; ^3^ Conseil Départemental des Alpes Maritimes Nice France

**Keywords:** community shift, northwestern Mediterranean, thermophilous teleost fish

## Abstract

The bastard grunt *Pomadasys incisus* (Bowdich, 1825), an Atlantic teleost, has recently been recorded at multiple sites along the French Ligurian Sea. Three juveniles were captured in Villepey Lagoon, while several groups of sub‐adult and adult individuals were observed at Cagnes‐sur‐Mer and Monaco between 2020 and 2024. These records indicate a shift from occasional sightings to a perennial presence, likely linked to ongoing Mediterranean Sea warming favouring this warm‐adapted species.

The bastard grunt *Pomadasys incisus* (Bowdich 1825) originates from the Atlantic. Entering naturally through the strait of Gibraltar, it is also considered as native to the Mediterranean. *Pomadasys incisus* is a coastal demersal species found in both marine and brackish environments, typically over sandy or muddy bottoms at depths up to 100 m (Kapiris et al., 2008). It can also be found in rocky areas and within seagrass meadows. It feeds predominantly on benthic invertebrates. Spawning occurs mainly in late spring and summer, with pelagic egg and larvae stage during approximately 3–4 weeks, and individuals reach sexual maturity at relatively small body sizes: 15–18 cm total length (TL) (Froese & Pauly, 2024). While its presence in the Mediterranean basin is documented at least since 1840 (Bodilis et al., 2013 and references therein; Doumpas et al., 2020), early but numerous sightings of the species have been recorded along the Gulf of Lion in 2005 (Pastor et al., 2008), allowing to consider the species as well established and later in 2011 in the French Ligurian Sea (Bodilis et al., 2013).

Between 2020 and 2024, multiple records provide evidence for the settlement of a perennial population of *Pomadasys incisus* along the French Ligurian Sea at three locations: the Gulf of Fréjus, Cagnes‐sur‐Mer, and Monaco (Figure 1a). This study aims to update the status of *P. incisus* population in the region by documenting its transition from sporadic occurrences to an established population and to discuss the potential link with ongoing Mediterranean Sea warming.

**FIGURE 1:**
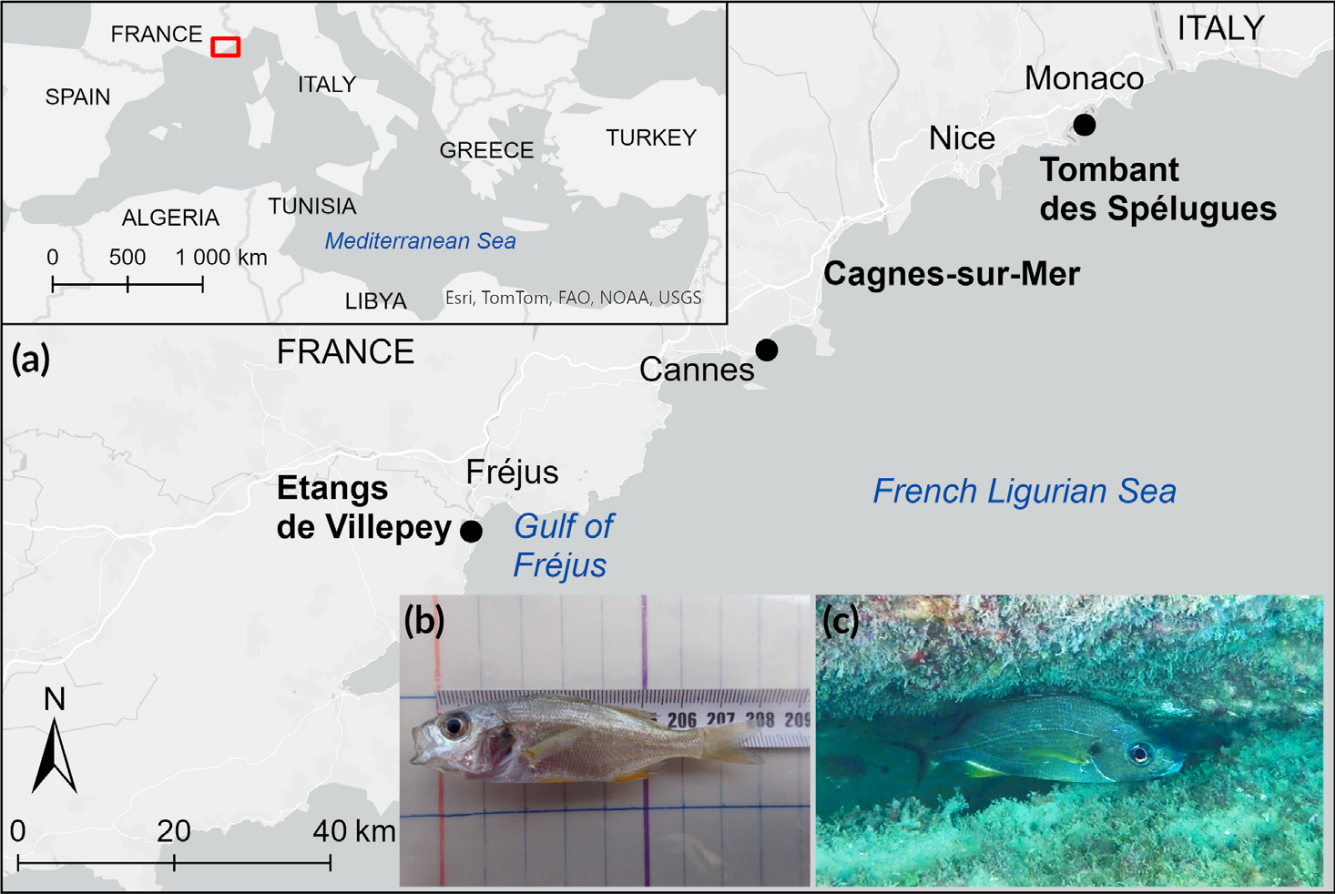
(a) Location of the three *Pomadasys incisus* sightings in French Ligurian Sea. (b) Individuals caught with a fyke net in Etang de Villepey in June 2024 ©David Héritier. (c) Individual spotted in Cagnes‐sur‐mer artificial reef area in December 2020 ©Christophe Serre.

Three juvenile individuals were captured in the Villepey Lagoon (Gulf of Fréjus) during an experimental fishing survey using a fyke net (Figure 1b). Additional records were obtained through underwater visual censuses: a school of 8–10 adult individuals (15–25 cm TL) observed on artificial reefs at Cagnes‐sur‐Mer in December 2020 and February 2022 (Figure 1c), and six sub‐adult individuals (12 cm TL) recorded on shallow rocky reefs in Monaco in September 2024 (*Tombant des Spélugues*) (Table 1).

**TABLE 1:** Information related to *Pomadasys incisus* sightings (number of individuals, total length, depth, method of sampling).

Location	Date	Nb. of ind. and TL (cm)	Depth (m)	Habitat	Sampling method	Authors
Etang de Villepey (Gulf of Fréjus)	4 June 2024	2 of 6.6 and 7.0 cm	1 m	Brackish lagoon	Fyke net	DH
	7 June 2024	1 of 7.5 cm	1 m			DH
Cagnes‐sur‐mer (Bay of Nice)	16–17 December 2020	⁓10 of 15–25 cm	10–15 m	Artificial reef on sandy mud	UVC	CS
	2 February 2022	8 of 18–22 cm				CS, YB
Tombant des Spélugues (Monaco)	6 September 2024	6 of 12 cm	19 m	Shallow rocky reefs	UVC	RB

Abbreviations: CS, Christophe Serre; DH, David Héritier; Nb. of ind., number of individuals; RB, Romain Bricout; TL, total length; UVC, underwater visual census; YS, Yann Strebler.

*Pomadasys incisus* is diagnosed by an oblong, laterally compressed body with a short snout (≈ eye diameter) and small mouth, the maxilla not reaching the anterior margin of the eye. The dorsal fin has XII spines +15–16 soft rays; the anal fin has III spines +11–13 soft rays; 27 vertebrae; a chin with two pores and a median pit; the caudal fin is forked. A dark blotch on the upper opercle further supports identification (Froese & Pauly, 2024; Figure 1b,c).

Following the previously reported punctual and localized record of *Pomadasys incisus* in the French Ligurian Sea by Bodilis et al. (2013), we report here new observations indicating the presence of an established population. Prior to Bodilis et al. (2013), the species had been recorded in 1991 along the Italian Ligurian coast, near the present study area (Gavagnin et al., 1994). The three juvenile individuals captured in Villepey Lagoon (up to 7.5 cm TL) likely indicate the proximity of a breeding site, confirming the species' perennial presence in the region. Since 2022, the collaborative GBIF database has reported several *P. incisus* records along the French coast, with four, three, two and two sightings in 2022, 2023, 2024 and 2025, respectively. These records are mainly concentrated in the French Riviera and Corsica (GBIF, 2026). Although *P. incisus* was rarely reported prior to this period, this apparent increase may also partly reflect changes in observation effort and reporting intensity.

The northward expansion of *P. incisus* is attributed to ongoing warming of the Mediterranean Sea (Bodilis et al., 2013; Villegas‐Hernández et al., 2014). These records support additional evidence of ongoing changes in teleost assemblages and the establishment of thermophilous species in the northwestern Mediterranean (Astruch et al., 2022; Azzola et al., 2024). However, Villegas‐Hernández et al. (2014) evidenced that even installed populations might exhibit different life traits (e.g., lower egg size and quality but higher abundance) than in meridional (warmer) areas.

Compared with other more conspicuous thermophilous teleosts (e.g., *Thalassoma pavo* (Linnaeus, 1758), *Sparisoma cretense* (Linnaeus, 1758), *Mycteroperca rubra* (Bloch, 1793); Azzola et al., 2024; Domingues et al., 2008), *P. incisus* is more low profile, inhabits sites less frequented by recreational divers, and can be easily misidentified (e.g., with Sparidae species). This likely explains its low number of records and highlights the need for enhanced monitoring of thermophilous teleosts, both native and non‐indigenous. Coordinated citizen‐science programs, as implemented, for example, in the eastern Mediterranean (Doumpas et al., 2020; Giovos et al., 2019), could help cover large areas and sight new species earlier. Furthermore, quantitative data from experimental fishing or standardized underwater visual census could provide valuable information on size structure and improve understanding of ongoing shifts in fish assemblages.

## AUTHOR CONTRIBUTIONS

All authors have significantly contributed to this work. **Astruch Patrick** contributed to conception, data curation, writing and editing. **Héritier David** contributed to field investigation, data collection and review. **Serre Christophe** contributed to field investigation and data collection. **Strebler Yann** contributed to field investigation, data collection and review. **Schohn Thomas** contributed to field investigation, data collection and review. **Belloni Bruno** contributed to field investigation, data collection and review. **Le Diréach Laurence** contributed to field investigation, data collection and review.

## FUNDING INFORMATION

Field investigations in Monaco were funded by the Monaco Environment Agency.

## Data Availability

The data that support the findings of this study are available on request from the corresponding author. The data are not publicly available due to privacy or ethical restrictions.

## References

[jfb70488-bib-0001] Astruch, P. , Belloni, B. , Rouanet, E. , Schohn, T. , Harmelin‐Vivien, M. , Harmelin, J. G. , & Boudouresque, C. F. (2022). Dominance of *Scorpaena maderensis* among scorpaenids of littoral rocky reefs in Corsica (NW Mediterranean): Further evidence of Mediterranean Sea warming. Journal of Fish Biology, 100(2), 601–604. 10.1111/jfb.14972 34873704

[jfb70488-bib-0002] Azzola, A. , Bianchi, C. N. , Merotto, L. , Nota, A. , Tiralongo, F. , Morri, C. , & Oprandi, A. (2024). The changing biogeography of the Ligurian Sea: Seawater warming and further records of southern species. Diversity, 16(3), 159. 10.3390/d16030159

[jfb70488-bib-0003] Bodilis, P. , Crocetta, F. , Langeneck, J. , & Francour, P. (2013). The spread of an Atlantic fish species, *Pomadasys incisus* (Bowdich, 1825) (Osteichthyes: Haemulidae), within the Mediterranean Sea with new additional records from the French Mediterranean coast. Italian Journal of Zoology, 80(2), 273–278. 10.1080/11250003.2012.730555

[jfb70488-bib-0004] Domingues, V. S. , Alexandrou, M. , Almada, V. C. , Robertson, D. R. , Brito, A. , Santos, R. S. , & Bernardi, G. (2008). Tropical fishes in a temperate sea: Evolution of the wrasse *Thalassoma pavo* and the parrotfish *Sparisoma cretense* in the Mediterranean and the adjacent Macaronesian and Cape Verde archipelagos. Marine Biology, 154(3), 465–474. 10.1007/s00227-008-0941-z

[jfb70488-bib-0005] Doumpas, N. , Tanduo, V. , Crocetta, F. , Giovos, I. , Langeneck, J. , Tiralongo, F. , & Kleitou, P. (2020). The bastard grunt *Pomadasys incisus* (Bowdich, 1825) (Teleostei: Haemulidae) in Cyprus (eastern Mediterranean Sea)—a late arrival or just a neglected species? Biodiversity Data Journal, 8, e58646. 10.3897/BDJ.8.e58646 33281474 PMC7704525

[jfb70488-bib-0006] Froese, R. , & Pauly, D. (Eds.). (2024). Pomadasys incisus . In: FishBase. Retrieved January 28, 2026, from https://www.fishbase.se

[jfb70488-bib-0007] Gavagnin, P. F. , Garibaldi, F. , & Relini, M. (1994). Segnalazione di *Pomadasys incisus* (Bowdich) (Osteichthyes, Haemulidae) in acque italiane. Biologia Marina Mediterranea, 1, 285–286.

[jfb70488-bib-0008] GBIF . (2026). *Pomadasys incisus* (Bowdich, 1825). In GBIF Secretariat (2023). GBIF Backbone Taxonomy. Checklist dataset. 10.15468/39omei GBIF.org 12 January 2026.

[jfb70488-bib-0009] Giovos, I. , Kleitou, P. , Poursanidis, D. , Batjakas, I. , Bernardi, G. , Crocetta, F. , Doumpas, N. , Kalogirou, S. , Kampouris, T. E. , Keramidas, I. , Langeneck, J. , Maximiadi, M. , Mitsou, E. , Stoilas, V.‐O. , Tiralongo, F. , Romanidis‐Kyriakidis, G. , Xentidis, N.–. J. , Zenetos, A. , & Katsanevakis, S. (2019). Citizen‐science for monitoring marine invasions and stimulating public engagement: A case project from the eastern Mediterranean. Biological Invasions, 21(12), 3707–3721. 10.1007/s10530-019-02083-w

[jfb70488-bib-0010] Kapiris, K. , Kallias, E. , & Conides, A. (2008). Preliminary biological data on *Pomadasys incisus* (Osteichthyes: Haemulidae) in the Aegean Sea, Greece. Mediterranean Marine Science, 9, 53–62.

[jfb70488-bib-0011] Pastor, J. , Astruch, P. , Prats, E. , Dalias, N. , & Lenfant, P. (2008). Premières observations en plongée de *Pomadasys incisus* (Haemulidae) sur la côte catalane française. Cybium, 32(2), 185–186.

[jfb70488-bib-0012] Villegas‐Hernández, H. , Lloret, J. , & Muñoz, M. (2014). Climate‐driven changes in life‐history traits of the bastard grunt (*Pomadasys incisus*) in the north‐western Mediterranean. Mediterranean Marine Science, 16(1), 21–30. 10.12681/mms.951

